# Anxiety Sensitivity in School Attending Youth: Exploratory and Confirmatory Factor Analysis of the 18-Item CASI in a Multicultural South African Sample

**DOI:** 10.3389/fpsyg.2015.01996

**Published:** 2016-01-07

**Authors:** Lindi Martin, Martin Kidd, Soraya Seedat

**Affiliations:** ^1^Department of Psychiatry, Stellenbosch University Cape Town, South Africa; ^2^Centre for Statistical Consultation, Department of Statistics and Actuarial Sciences, Stellenbosch UniversityCape Town, South Africa

**Keywords:** Childhood Anxiety Sensitivity Index (CASI), exploratory factor analysis, confirmatory factor analysis, gender, adolescents, South Africa

## Abstract

Anxiety sensitivity (AS) is a risk factor for the development of anxiety disorders in youth. To date, the applicability of the Childhood Anxiety Sensitivity Index (CASI) in youth from a low or middle income country (LMIC) setting on the African continent has not been assessed. A representative sample of 1149 secondary school learners from 29 schools in Cape Town, South Africa, participated in the study. Participants completed the CASI on a single occasion. One-, two-, and four-factor models of the CASI were assessed. A one-factor solution that comprised items predominantly represented by physical concerns appeared to provide the best fit to our data, however, relatively low variance (26%) was explained. Subsequent item deletion resulted in a 9-item ‘physical concerns’ factor that showed good construct reliability (0.83) but also explained a low amount of variance (35%). In terms of gender, a one-factor model provided the best fit, however, low variance was explained (i.e., 25%). Configural, metric and scalar invariance of the CASI by gender was determined. Our results suggest that the 18-item CASI is not applicable to our target population and may require adaptation in this population; however, replication of this study in other multicultural adolescent samples in South Africa is first needed to further assess the validity of the AS construct as measured by the CASI.

## Introduction

Anxiety sensitivity (AS) is an established temperamental trait and vulnerability factor associated with fearfulness and anxiety pathology in both adults and youth (Reiss et al., 1986; McLaughlin et al., 2007; Naragon-Gainey, 2010; Viana and Gratz, 2012; Olthuis et al., 2014). AS is defined as the fear of anxiety-related bodily sensations and symptoms (e.g., feeling nervous, dizzy or shaky; heart beating fast; stomach growling) based on the belief that these somatic sensations have negative or even catastrophic physical, psychological or social consequences (e.g., being physically ill; psychological incapacitation; social embarrassment) (Reiss and McNally, 1985; Reiss, 1991). These beliefs or expectations are thought to increase an individual’s pre-existing anxiety (Reiss, 1991).

In children and adolescents, AS has been shown to be strongly and significantly associated with measures of anxiety symptoms and anxiety disorder subtypes (McLaughlin et al., 2007; Essau et al., 2010), including panic attack symptomatology and distress levels due to panic symptomatology (Lau et al., 1996). Furthermore, AS has been shown to prospectively predict the development of anxiety symptoms in children, adolescents (Schmidt et al., 2010) and young adults (Schmidt et al., 2008), including the incidence of spontaneous panic attacks (in those without panic histories) and overall Axis I diagnoses, in non-clinical young adults (Schmidt et al., 2006). It has been established that individuals diagnosed with anxiety disorders report significantly greater levels of AS than non-clinical controls (Olatunji and Wolitzky-Taylor, 2009), signifying that heightened AS is a risk factor for anxiety symptoms and disorders.

In adults, AS is commonly measured using the Anxiety Sensitivity Index (ASI; Peterson and Reiss, 1987), a 16-item, self-report questionnaire rated on a 5-point scale ranging from 0 to 4 (*‘very little’* to *‘very much’*). The 18-item Childhood Anxiety Sensitivity Index (CASI; Silverman et al., 1991), typically used to measure AS in children and adolescents, was derived from the ASI items but was modified to be age-appropriate, relevant and readily understandable to children (Silverman et al., 1991). Responses on the CASI are rated on a 3-point scale ranging from 1 to 3 (*‘none’* to *‘a lot’*).

Results from early factor analytic studies on AS in adult samples (e.g., spider phobic college students, adult psychiatric outpatients and college students), as measured by the ASI, suggested that the ASI is unifactorial in nature and thus consists of a single factor (Reiss et al., 1986; Taylor et al., 1991). Comparable findings were documented in Spanish treatment seekers (Sandin et al., 1996) and in Native Americans (Norton et al., 2004). More recently, a meta-analysis of AS indicated that AS in adults is multidimensional, consisting of three distinct, yet intercorrelated factors (Olatunji and Wolitzky-Taylor, 2009) that are hierarchically arranged and load on a single higher-order factor (Zinbarg et al., 1997). The three lower-order factors comprise (1) fear of physical symptoms, (2) fear of publicly observable anxiety symptoms and (3) fear of cognitive dyscontrol (Olatunji and Wolitzky-Taylor, 2009). That said, some studies have found that the ASI is comprised of two (e.g., Schmidt and Joiner, 2002) or even four (e.g., Carter et al., 1999) underlying factors.

Results from studies that have explored the factor structure of the CASI in children and adolescents appear relatively inconsistent. In one of the first of such studies, using the 18-item CASI in a sample of clinical and non-clinical children and pre-adolescents, Silverman et al. (1999) found support for a hierarchical multidimensional model in which three or four-factors were present, of which two were robust, namely, physical and mental incapacitation concerns. Subsequent confirmatory factor analysis by Silverman et al. (2003), in samples of non-clinical children and adolescents, based on results from past factor analytic studies, supported a hierarchical factor model for AS, due to a strong general factor. Furthermore, there was evidence for four lower-order factors (i.e., disease concerns, unsteady concerns, mental incapacitation concerns, and social concerns) that fit the data well (Silverman et al., 2003). That said, a three-factor solution, as found in the adult literature, has also commonly been documented in non-clinical samples of children and adolescents using the CASI (van Widenfelt et al., 2002; McLaughlin et al., 2007). Given the aforesaid, the factor structure of the CASI is still essentially questionable and may potentially vary according to sample characteristics.

In terms of gender, a number of studies that have compared CASI factor models across gender have shown that AS appears similar in structure for males and females. For example, Walsh et al. (2004) reported similar structures across gender for their three-factor lower-order models in a large school-attending sample of children and adolescents. Similarly, Wright et al. (2010) found that the fit for a three-factor model was similar across gender in a comparable sample and Silverman et al. (2003) found support for factorial invariance across gender for their four-factor lower-order models in both clinical and non-clinical samples of children and adolescents.

An important finding, when examining the AS construct in youth from non-Caucasian ethnic groups, is that AS appears to manifest in ways different to that commonly found in Caucasian samples of children, adolescents and young adults (Carter et al., 1999; Lambert et al., 2004). For example, Lambert et al. (2004) reported higher mean AS scores in their sample of 144 African American fourth- and fifth-grade students, than is commonly found in studies of non-clinical Caucasian children and adolescents. Furthermore, they found that the three- and four-factor higher-order model as proposed by Silverman et al. (1999) did not provide a good fit, but rather that a two-factor model best fit their data. The two-factors were ‘physical concerns’ and ‘mental incapacitation,’ with little support for a social concerns or control factor. In addition, contrary to the general trend in Caucasian samples, they found no gender difference in levels of AS in their sample. Similarly, Carter et al. (1999), using the ASI in a sample of 221 African American college students, found that the commonly agreed upon three factor model by Zinbarg et al. (1997) of ‘physical concerns,’ ‘mental incapacitation concerns,’ and ‘social concerns,’ did not provide a good fit to their data. Rather, they found support for a four-factor model, with the composition of factors seemingly different from that commonly found among Caucasian samples (Carter et al., 1999). Lambert et al. (2004) indicated that different factor structures may reflect cross-ethnic dissimilarities in the AS construct.

To date, the applicability of the CASI, a measure developed in the United States, has not been assessed in South African youth. As such, the current study examined the construct of AS in a representative sample of predominantly Black and mixed-race secondary school learners from Cape Town, South Africa. The primary aim of the study was to assess the factorial validity of the CASI by firstly, conducting an exploratory factor analysis (EFA) to determine the underlying factor structure of the CASI in our sample overall and by gender and secondly, by performing confirmatory factor analyses (CFA) on the sample overall and by gender to examine the fit of the models that emerged from the EFA. An investigation into the factorial validity of the CASI will aid our understanding of the etiology of AS in a representative sample of predominantly non-Caucasian secondary school attenders, in a lower-income, multi-cultural setting.

## Materials and Methods

### Participants and Procedure

Permission to access secondary schools in Cape Town and to conduct the study was provided by the Western Cape Education Department (WCED) and the Health Research Ethics Committee at Stellenbosch University, respectively.

The education districts of Cape Town, as indicated by the WCED, were stratified according to those representing urban education districts (i.e., Metro North, Metro South, Metro East, and Metro Central). Schools (*n* = 29) were then randomly sampled from each of these districts to obtain a sample representative of urban public secondary schools in Cape Town. Schools identified, as described above, were approached to participate in the study and those that agreed to participate were requested to provide the names of all learners from grades 8 to 12. Thereafter, a sample of 20 learners per grade, per school, was randomly selected so as to ensure a representative sample of secondary school learners from Cape Town. Written informed consent was obtained from parents or guardians and written assent was obtained from the learners themselves. Study questionnaires were completed at the schools on a single occasion.

The resulting sample of learners comprised 1149 youths aged between 13 and 23 years (*M* = 16.24, *SD* = 1.95). The majority of the sample was classified as adolescents aged from 13 to 18 years (995/1149, 86.6%). The mean level of education was grade 9 and ranged between grades 8 and 12. Over half the sample consisted of girls (689/1149, 59.97%). The vast majority of the sample identified themselves as black (68.9%), followed by mixed-race (27.7%).

### Measure

The *CASI* (Silverman et al., 1991) is an 18-item self-report questionnaire designed for use with school-age children and adolescents. The CASI measures the fear of anxiety symptoms on a 3-point Likert-type scale by asking participants to rate the extent to which they believe the experience of anxiety will result in negative consequences, comprising physical, psychological and social concerns. The CASI yields a total score by summing the 18 items and has a range of 18–54 with higher scores reflecting higher levels of AS. Silverman et al. (1991) reported adequate internal consistency and reliability for the CASI in their sample of clinical and non-clinical, primarily Caucasian, children and adolescents. In the current study, for the total sample, the CASI had good internal consistency (*α* = 0.81), with Cronbach’s alphas of 0.80 and 0.81 for boys and girls, respectively. Corrected item-total correlation values ranged from 0.207 to 0.509. Three of eighteen items (i.e., items 1, 5, and 17) (see **Table 1**) displayed values that were below the commonly accepted level of 0.3 (Pallant, 2007). However, examination of the item-total statistics (i.e., Cronbach’s alpha if item deleted) indicated that removal of any item would not have improved the reliability of the CASI and thus all 18 CASI items were retained.

**Table 1:** 18-item CASI: corrected item-total correlation values (CITC), item response frequencies, mean, skewness and kurtosis.

		Item responses [*n*(%)]					
					
CASI items	CITC	None	Some	A lot	Mean (*SD*)	Skewness	Kurtosis
Item 1	0.207	262 (23)	718 (62)	169 (15)	1.92 (0.61)	0.041	-0.320
Item 2	0.365	501 (44)	432 (38)	216 (19)	1.75 (0.75)	0.441	-1.113
Item 3	0.502	415 (36)	452 (39)	282 (25)	1.88 (0.77)	0.201	-1.292
Item 4	0.436	404 (35)	378 (33)	367 (32)	1.97 (0.82)	0.059	-1.506
Item 5	0.227	139 (12)	321 (28)	689 (60)	2.48 (0.70)	-0.979	-0.358
Item 6	0.470	249 (22)	493 (43)	407 (35)	2.14 (0.74)	-0.227	-1.165
Item 7	0.340	362 (32)	450 (39)	337 (29)	1.98 (0.78)	0.038	-1.355
Item 8	0.405	418 (36)	478 (42)	253 (22)	1.86 (0.75)	0.242	-1.196
Item 9	0.509	335 (29)	428 (37)	386 (34)	2.04 (0.79)	-0.079	-1.398
Item 10	0.466	279 (24)	402 (35)	468 (41)	2.16 (0.79)	-0.300	-1.339
Item 11	0.391	299 (26)	542 (47)	308 (27)	2.01 (0.73)	-0.012	-1.106
Item 12	0.422	214 (19)	403 (35)	532 (46)	2.28 (0.76)	-0.508	-1.098
Item 13	0.303	586 (51)	428 (37)	135 (12)	1.61 (0.69)	0.693	-0.677
Item 14	0.509	353 (31)	472 (41)	324 (28)	1.97 (0.77)	0.043	-1.300
Item 15	0.380	703 (61)	326 (28)	120 (10)	1.49 (0.68)	1.036	-0.174
Item 16	0.402	323 (28)	564 (49)	262 (23)	1.95 (0.71)	0.077	-1.024
Item 17	0.240	242 (21)	518 (45)	389 (34)	2.13 (0.73)	0.203	-1.109
Item 18	0.472	402 (35)	450 (39)	297 (26)	1.91 (0.77)	0.159	-1.319


*Standard Error of Skewness = 0.072; Standard Error of Kurtosis = 0.144.*

### Statistical Analyses

A very small amount of missing data was observed in the CASI items due to the strict procedures followed during the data collection phase, with all data being collected at schools and subsequently all study questionnaires being examined for missing data at the respective schools, at the time of questionnaire completion. As such, a maximum of 1.22% of missing values was evident in the CASI items. Missing data were replaced by means of the k-nearest neighbor imputation method.

Descriptive statistics were computed for demographic data, with variables of interest including age, gender, and ethnicity. Frequencies of responses to the 18 CASI items were reported, along with item means (and *SD*’s), skewness and kurtosis. Differential item functioning (DIF) analyses, using the lordif package (Choi et al., 2011), was conducted using iterative hybrid ordinal logistic regression (IRT DIF) to determine whether males and females were consistent in how they interpreted and endorsed CASI items.

Examination of the underlying factor structure of the CASI for the total sample and by gender was determined by means of EFA using principal components analysis (PCA) with oblique rotation. In addition to conducting Pearson correlations, polychoric correlations were calculated and it was determined that results were similar to those found for Pearson correlations. Factors retained were based on results of (1) parallel analysis, (2) Cattell’s scree test and (3) a Kaiser’s eigenvalue greater than one. A cut-off of 0.3, in conjunction with clinical judgment, was used as a reference to indicate salient item loadings. Thereafter, CFA, using Lisrel version 8.8, was conducted to test the suitability of the models that emerged from the EFA. Robust maximum-likelihood (RML) was used as the method of estimation; a useful estimation method employed to deal with possible non-normal data. All variables were treated as continuous variables. Multiple fit indices were used to indicate how well the proposed models fitted the data. The following indices were used in the present study: (1) the Root Mean Square Error of Approximation (RMSEA; should be 0.05 or lower); (2) the Standardized Root Mean Square Residual (SRMR; should be 0.08 or less); (3) the Comparative Fit Index (CFI; should be closer to 1); (4) the Goodness of Fit Index (GFI; should be 0.90 or higher); and (5) the Adjusted Goodness of Fit Index (AGFI; should be 0.90 or higher). To assist with model comparison and selection for analysis on the total sample, we reported the following information criteria: (1) the Akaike Information Criterion (AIC; lower values are preferred) and (2) the Consistent Akaike Information Criterion (CAIC, lower values are preferred). Structural equation modeling provided an indication of the strength of the relationship between CASI items and proposed factors and between individual factors in each model. As such, the total sample of 1149 participants was randomly split into two. The first cohort, on which the EFA was conducted, consisted of 30% of the sample (345/1149) and the second cohort, on which CFA was run, consisted of the remaining 70% (804/1149). Similarly, EFA was conducted on 30% of males and females and CFA was conducted on the remaining 70% of males and females, using the above named procedures. Finally, we examined measurement invariance of the CASI across genders, using multigroup CFA with RML estimation, to assess configural (i.e., whether the same model structure holds across genders) and metric (i.e., whether factor loadings are similar across genders) invariance. In terms of assessing scalar invariance (i.e., whether intercepts are equal across genders), maximum likelihood estimation was employed.

## Results

### Descriptive Statistics: 18-Item CASI

The frequencies of responses to the individual CASI items as well as the item means, skewness and kurtosis values are presented in **Table 1.**

### DIF by Gender

Results indicated that 2 of the 18 items had DIF, namely, items 1 and 3 [i.e., ‘I don’t want other people to know when I feel afraid’ (*p* = 0.023) and ‘It scares me when I feel shaky’ (*p* = 0.001)]. The magnitude of DIF was calculated for the two items and was found to be negligible (Nagelkerke *R*^2^ < 0.035).

### EFA of the CASI for the Total Sample

**FIGURE 1:**
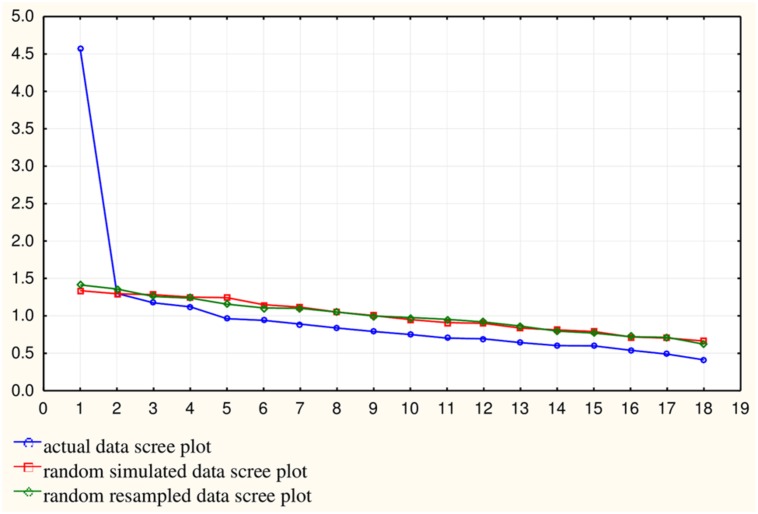
**Scree plot with parallel analysis results.** It indicates the number of factors to be retained. The blue scree plot represents the scree plot from the actual data while the red and green scree plots represent randomly simulated data with the same dimensions as the actual data. This figure clearly indicates one-factor and the possibility of two factors, as the first factor lies clearly above the random scree plots and the second factor lies directly on the random scree plots.

Factors retained were based on results of the parallel analysis, Cattell’s scree test and a Kaiser’s eigenvalue greater than one. Parallel analysis indicated a one-factor model or the possibility of a two-factor model, whereas the scree plot indicated a two-factor model. Using an eigenvalue greater than one rule, four-factors were suggested. As such, we explored one-, two-, and four-factor models. See **Figure 1** depicting the scree plot and results of the parallel analysis.

#### One-Factor Model

The one-factor in the model explained 25.35% of the variance. Loadings were relatively low, all below 0.65. All items with loadings above 0.5 were related to physical concerns (see **Table 2**).

**Table 2:** EFA one-factor model.

Item	Description of CASI items	Loading
1	Don’t want other people to know when I am afraid.	0.42
2	When I can’t keep mind on schoolwork, worry I going crazy.	0.42
3	Scares me when I feel shaky.	0.64
4	Scares me when I feel like I am going to faint.	0.55
5	Important for me to stay in control of my feelings.	0.28
6	Scares me when my heart beats fast.	0.57
7	Embarrasses me when my stomach growls.	0.49
8	Scares me when feels like going to throw up.	0.49
9	When my heart beats fast, worry something wrong with me.	0.62
10	Scares me when having trouble getting my breath.	0.55
11	When my stomach hurts, worry that I might be really sick.	0.51
12	Scares me when can’t keep my mind on schoolwork.	0.48
13	Other kids can tell when I feel shaky.	0.47
14	Unusual feelings in my body scare me.	0.59
15	When I am afraid, worry that I might be crazy.	0.47
16	Scares me when I feel nervous.	0.49
17	Don’t like to let my feelings show.	0.27
18	Funny feelings in my body scare me.	0.58


#### Two-Factor Model

Combined, the two-factors in the model explained 32.57% of the total variance, with the first and second factors accounting for 25.35% (eigenvalue = 4.56) and 7.22% (eigenvalue = 1.3) of the variance, respectively. The first factor (items 9, 11, 3, 2, 13, 15, 6, 18, 7, 8, 10, 16, 14, and 12) was labeled ‘physical and psychological concerns’ and consisted of a combination of items representing both physical and psychological concerns (see **Table 3**). The second factor was labeled ‘social and control concerns’ and consisted predominantly of items representing social concerns (i.e., items 1, 5, and 17). Item 4 (‘It scares me when I feel like I am going to faint’), which loaded onto the second factor, represented an item also commonly associated with factors labeled in the literature as an ‘unsteady concern’ (Silverman et al., 2003), a ‘control concern’ (Silverman et al., 1999) and a ‘physical concern’ (Silverman et al., 2003). The correlation between the two-factors was 0.30.

**Table 3:** EFA two-factor model.

Item	Description of CASI items	Factor 1	Factor 2
9	When my heart beats fast, worry something wrong with me.	0.69	
11	When my stomach hurts, worry that I might be really sick.	0.62	
3	Scares me when I feel shaky.	0.60	
2	When I can’t keep mind on schoolwork, worry I going crazy.	0.57	
13	Other kids can tell when I feel shaky.	0.56	
15	When I am afraid, worry that I might be crazy.	0.55	
6	Scares me when my heart beats fast.	0.54	
18	Funny feelings in my body scare me.	0.48	
7	Embarrasses me when my stomach growls.	0.46	
8	Scares me when feels like going to throw up.	0.43	
10	Scares me when having trouble getting my breath.	0.42	
16	Scares me when I feel nervous.	0.40	
14	Unusual feelings in my body scare me.	0.39	
12	Scares me when can’t keep my mind on schoolwork.	0.38	
17	Don’t like to let my feelings show.		0.74
1	Don’t want other people to know when I feel afraid.		0.64
5	Important for me to stay in control of my feelings.		0.41
4	Scares me when I feel like I am going to faint.		0.38


*Factor 1: physical and psychological concerns; Factor 2: social and control concerns.*

#### Four-Factor Model

The four-factor model, indicated by eigenvalues greater than 1, explained 45.31% of the total variance. The first factor accounted for 25.35% (eigenvalue = 4.56) of the variance; the second factor explained 7.22% (eigenvalue = 1.30) and the third and fourth factors explained 6.52% (eigenvalue = 1.17) and 6.22% (eigenvalue = 1.12) of the variance, respectively. The first factor was labeled ‘mental incapacitation and physical concerns’ (items 15, 16, 18, and 9) (see **Table 4**). The second factor was labeled ‘social concerns’ (items 17 and 1), and the third factor and fourth factors were labeled ‘control concerns’ (items 5, 12, and 2) and ‘physical concerns’ (items 10, 8, 14, 7, 11, 4, 13, 6, and 3), respectively. Correlations between the four-factors were relatively modest and ranged from 0.05 to 0.37.

**Table 4:** EFA four-factor model.

Item	Description of CASI items	Factor 1	Factor 2	Factor 3	Factor 4
15	When I am afraid, worry that I might be crazy.	0.76			
16	Scares me when I feel nervous.	0.50			
18	Funny feelings in my body scare me.	0.50			
9	When my heart beats fast, worry something wrong with me.	0.48			
17	Don’t like to let my feelings show.		0.74		
1	Don’t want other people to know when I feel afraid.		0.63		
5	Important for me to stay in control of my feelings.			0.74	
12	Scares me when can’t keep my mind on schoolwork.			0.65	
2	When I can’t keep mind on schoolwork, worry I going crazy.			0.52	
10	Scares me when having trouble getting my breath.				0.78
8	Scares me when feels like going to throw up.				0.62
14	Unusual feelings in my body scare me.				0.62
7	Embarrasses me when my stomach growls.				0.45
11	When my stomach hurts, worry that I might be really sick.				0.43
4	Scares me when I feel like I am going to faint.				0.42
13	Other kids can tell when I feel shaky.				0.40
6	Scares me when my heart beats fast.				0.39
3	Scares me when I feel shaky.				0.36


*Factor 1: mental incapacitation and physical concerns; Factor 2: social concerns; Factor 3: control concerns; Factor 4: physical concerns.*

### CFA of the CASI for the Total Sample

Confirmatory factor analyses was conducted to assess the model fit for two- and four-factor models as well as a one-factor model. See **Table 5** for fit indices for these models. Results indicated acceptable goodness of fit indices for the three models, particularly the one- and four-factor models. Similarly, AIC and CAIC values suggested that the one- and four-factor models were more suitable than the two-factor model and as such, we report further on the one- and four-factor CFA models.

**Table 5:** Fit indices and information criteria for the one-, two- and four-factor models.

	One factor	Two factors	Four Factors
*χ*2(df)*p*	381.38(135)^∗^	403.76(134)^∗^	342.41(129)^∗^
RMSEA	0.048	0.05	0.045
SRMR	0.056	0.044	0.053
CFI	0.97	0.95	0.97
GFI	0.98	0.95	0.98
AGFI	0.97	0.93	0.97
AIC	453.37	477.76	426.41
CAIC	658.20	688.27	665.37


*χ^2^, Satorra–Bentler Weighted Least Squares Chi-Square; *df*, Degrees of Freedom; RMSEA, Root Mean Square Error of Approximation; SRMR, Standardized Root Mean Square Residual; CFI, Comparative Fit Index; GFI, Goodness of Fit Index; AGFI, Adjusted Goodness of Fit Index; AIC, Akaike Information Criterion; CAIC, Consistent Akaike Information Criterion; ^∗^*p* < 0.001.*

#### Construct Reliability and Variance Extracted

With regard to the one-factor model, good construct reliability was evident (i.e., *α* = 0.85). That said, the variance extracted was relatively low (i.e., 26%). Examination of the item loadings between the CASI items and the individual factor showed that 11 of the 18 items were 0.5 and above and the remaining 7 were lower than 0.5 (see **Table 6**), with all loadings being significant (i.e., *t*-statistic >1.96). The majority of items with higher loadings were representative of items that were related to ‘physical concerns.’ Given these results, a one-factor model appears to provide the best fit, as previously suggested by the scree plot.

**Table 6:** Item loadings between CASI items and individual CASI factor from the one-factor CFA model.

Item	Description of CASI items	Item loading
1	*Don’t want other people to know when I am afraid.*	0.16
2	*When I can’t keep mind on schoolwork, worry I going crazy.*	0.49
3	Scares me when I feel shaky.	0.60
4	Scares me when I feel like I am going to faint.	0.54
5	*Important for me to stay in control of my feelings.*	0.28
6	Scares me when my heart beats fast.	0.62
7	*Embarrasses me when my stomach growls.*	0.38
8	Scares me when feels like going to throw up.	0.51
9	When my heart beats fast, worry something wrong with me.	0.66
10	Scares me when having trouble getting my breath.	0.59
11	*When my stomach hurts, worry that I might be really sick.*	0.48
12	Scares me when can’t keep my mind on schoolwork	0.56
13	*Other kids can tell when I feel shaky.*	0.36
14	Unusual feelings in my body scare me.	0.64
15	When I am afraid, worry that I might be crazy.	0.53
16	Scares me when I feel nervous.	0.50
17	*Don’t like to let my feelings show.*	0.29
18	Funny feelings in my body scare me.	0.59


*Items in italics reflect those with path coefficients lower than 0.5.*

In terms of the four-factor model, the Lisrel software flagged a ‘positive definite’ warning, indicating highly correlated factors and thus suggesting that less than four factors are evident.

#### Deletion of Items from the One-Factor CFA Model

The one-factor CFA model was re-examined in order to identify items for possible deletion in order to determine a subset of items suggestive of a definite underlying construct. The inclusion and exclusion of items was based on both the examination of item loadings and discriminating between items that made clinical sense to include and exclude. As the majority of items with high loadings were those that related to ‘physical concerns,’ and thus suggested that a ‘physical concerns’ scale was dominant, we included all ‘physical concerns’ items and excluded those items that related to ‘psychological,’ ‘social’ and ‘control concerns.’ The resulting ‘physical concerns’ scale included the following nine items: (1) item 3 (‘scares me when I feel shaky’), (2) item 4 (‘scares me when I feel like I am going to faint’), (3) item 6 (‘scares me when my heart beats fast’), (4) item 8 (‘scares me when feels like going to throw up’), (5) item 9 (‘when my heart beats fast, worry something wrong with me’), (6) item 10 (‘scares me when having trouble getting my breath’), (7) item 11 (‘when my stomach hurts, worry that I might be really sick’), (8) item 14 (‘unusual feelings in my body scare me’) and (9) item 18 (‘funny feelings in my body scare me’).

Subsequently, both EFA and CFA, using the above-mentioned nine items, were conducted on the test data. The EFA clearly indicated the presence of one-factor and the amount of variance explained was 35.48%, with item loadings ranging from 0.51 to 0.66. The subsequent CFA indicated that the model explained 35% of the variance and the construct reliability was 0.83. Item loadings ranged from 0.48 to 0.67. The following fit indices were determined: χ^2^(df) = 101.09(27), *p* < 0.001; RMSEA: 0.058; SRMR: 0.048; CFI: 0.98; GFI: 0.99; and AGFI: 0.98. These results indicate good fit indices for the 9-item one-factor CFA model; however, the variance explained by this one-factor model remained low. The 9-item scale had good internal consistency (*α* = 0.77).

### EFA and CFA of the CASI by Gender

For both males and females, parallel analysis and scree plot results indicated that a one-factor model was most suitable. The one-factor in the model explained 25.38% and 24.40% of the variance for males and females, respectively. Factor loadings ranged between 0.16 and 0.67 for males and between 0.31 and 0.61 for females (see **Table 7**).

**Table 7:** One-factor EFA and CFA: item loadings by gender.

	EFA item loadings		CFA item loadings	
		
CASI items	Males	Females	Males	Females
1	0.48	0.35	0.23	0.15
2	0.31	0.47	0.51	0.46
3	0.67	0.60	0.62	0.57
4	0.51	0.57	0.58	0.49
5	0.25	0.31	0.29	0.29
6	0.63	0.50	0.61	0.60
7	0.53	0.45	0.44	0.31
8	0.52	0.46	0.47	0.54
9	0.67	0.57	0.67	0.64
10	0.51	0.55	0.56	0.60
11	0.55	0.46	0.44	0.50
12	0.46	0.47	0.54	0.54
13	0.53	0.45	0.34	0.38
14	0.57	0.60	0.64	0.64
15	0.44	0.50	0.49	0.54
16	0.48	0.49	0.48	0.52
17	0.16	0.33	0.27	0.29
18	0.51	0.61	0.54	0.60


In terms of CFA by gender, the fit indices for the one-factor model for both males and females (see **Table 8**), were acceptable. With regard to the one-factor model, good construct reliability was evident for both males and females (i.e., 0.85). As with the one-factor CFA model for the total sample, the variance extracted by the one-factor CFA model by gender was relatively low (i.e., 25% for both males and females). Item loadings between the CASI items and the individual factor for the one-factor CFA model by gender ranged from 0.23 to 0.67 for males and from 0.15 to 0.64 for females (see **Table 7**), with all loadings being significant (i.e., *t*-statistic > 1.96).

**Table 8:** Fit indices for the one-factor EFA model by gender.

	Males	Females
χ^2^(df)*p*	381.38(135)^∗^	403.76(134)^∗^
RMSEA	0.048	0.050
SRMR	0.056	0.044
CFI	0.97	0.95
GFI	0.98	0.95
AGFI	0.97	0.93


*χ^2^, Satorra–Bentler Weighted Least Squares Chi-Square; *df*, Degrees of Freedom; RMSEA, Root Mean Square Error of Approximation; SRMR, Standardized Root Mean Square Residual; CFI, Comparative Fit Index; GFI, Goodness of Fit Index; AGFI, Adjusted Goodness of fit Index; ^∗^*p* < 0.001.*

### Measurement Invariance by Gender

Configural invariance of the CASI was tested by examining whether an unconstrained model (i.e., outer loadings estimated separately for males and females) provided an acceptable fit. The configural invariance model indicated a reasonable fit, as evidenced by the RMSEA (0.047), the *p*-value for test of close fit (0.75) and the GFI (0.96). Further, we tested the metric invariance of the CASI by assessing if factor loadings were similar across genders. The *p*-value for differences between the constrained (i.e., model fitted under the hypothesis of equal outer loadings between males and females) and unconstrained models was 0.11. Scalar invariance of the CASI was tested by comparing a constrained model (i.e., under the hypothesis of equal intercepts) with an unconstrained model (i.e., intercepts estimated separately). Results indicated no differences between the models (*p* = 0.32). Taken together, these results provide support for configural, metric and scalar invariance across genders.

## Discussion

As the CASI, a measure developed in the United States, has not been assessed in South African youth, the current study examined the construct of AS, as measured by the 18-item CASI, in a representative sample of predominantly Black and mixed-race secondary school learners in South Africa. We aimed to assess the factorial validity of the CASI by conducting EFA and subsequent CFA to assess the resulting models in our sample overall and by gender.

Exploratory factor analysis indicated that the CASI consisted of one, two, or four underlying factors. Subsequent results from the CFA suggested that, of the models, the one-factor solution provided the best fit to our data. High correlations between the latent variables in the four-factor CFA model were evident as a ‘positive-definite’ warning was flagged by the Lisrel software indicating that less than four-factors are evident. Despite our one-factor CFA model demonstrating good construct reliability (i.e., 0.85), it explained a relatively small amount of variance (26%). Furthermore, a number of the items (i.e., 7 of 18) had relatively low item loadings. These comprised items 1, 2, 5, 7, 11, 13, and 17. Items 2 and 11, however, had relatively higher item loadings that were just below 0.5 (i.e., 0.49 and 0.48, respectively). Items 1, 5, 7, 13, and 17 are items generally associated with social and control concerns (Silverman et al., 1999, 2003; Muris et al., 2001), with item 2 reflecting a psychological concern and item 11 reflecting a physical concern (Silverman et al., 1999, 2003). The remaining 11 items reveal concerns predominantly relating to physical concerns (or disease concerns) and also contain some items that reflect psychological concerns (or mental incapacitation concerns) [e.g., items 12 and 15]. Given that the one-factor model appeared to best fit our data, our findings indicate a lack of equivalence with models found in Western samples (Silverman et al., 1999, 2003; Muris et al., 2001; Lambert et al., 2004; Walsh et al., 2004).

In order to determine a subset of items suggestive of a definite underlying construct, a number of items from the one-factor CFA model were deleted and subsequent EFA and CFA analyses was conducted. Based on both high item loadings and clinical judgment, a 9-item ‘physical concerns’ factor was derived. The higher item loadings of the majority of ‘physical concerns’ items in the one-factor model suggest that the participants in this study may have better understood these items and thus answered them more accurately. That said, as with the 18-item one-factor CFA model, the 9-item model showed good internal consistency but the variance extracted remained low (i.e., 35%). There is agreement that the items that we included in our 9-item physical concerns measure is inclusive of all items previously found to be labeled as ‘physical concerns’ in previous factor analytic studies of AS (Silverman et al., 2003). Previous factor analytic studies have consistently revealed support for a robust ‘physical concerns’ factor, associated with the strongest item loadings (e.g., Silverman et al., 1999; Chorpita and Daleiden, 2000). In a sample of adults with anxiety disorders, Zinbarg et al. (2001) found that their ‘physical concerns’ subscale (vs. ‘social concerns’ and ‘mental incapacitation’ subscales) of the 16-item ASI (1) had the largest correlation to fear responses in two physiological challenges (i.e., hyperventilation and carbon monoxide) and (2) uniquely contributed to variance in fear ratings during these challenges whilst the other two subscales did not, suggesting that the ‘physical concerns’ subscale plays a key role in panic disorder (Zinbarg et al., 2001). Of note, the items that constitute the ‘physical concerns’ factor previously mentioned (i.e., Zinbarg et al., 2001), duplicate those included in our 9-item measure. The ‘physical concerns’ factor [based on the three-factor model of Zinbarg et al. (1997)], assessed in adolescents and young adults (Dehon et al., 2005), displayed the largest partial correlation with anxiety (i.e., controlling for depression), compared with the ‘social’ and ‘psychological concerns’ factors, and it would be useful to determine whether our 9-item ‘physical concerns’ measure reveals similar findings, within the South African context, in future studies in samples comparable to the current study.

In terms of gender, we found that a one-factor model was suited to both males and females, however, in line with our results of the EFA and CFA for the total sample, the one-factor CFA model applied to both genders demonstrated good construct reliability (i.e., 0.85) but explained a relatively low amount of variance (25%). Our results showed support for configural, metric and scalar invariance across gender, indicating that the CASI assesses the same construct in both males and females in our sample.

Overall, a low amount of variance was extracted by the constructs from the individual CFA models in the sample overall and by gender. A relatively high level of item difficulty and complexity (e.g., the content of the questions and how they are phrased), resulting in the possible misinterpretation of the CASI items, may have influenced participants’ responses on the CASI and may have thus contributed to our findings (i.e., relatively low factor loadings and low variance extracted). In sum, given our results, the applicability of both the 18-item CASI as well as the 9-item factor derived from the original measure has proven limited in the current sample. The 9-item ‘physical concerns’ factor derived from the 18-item measure taps solely into the physical symptoms characteristic of the AS construct, a construct that the majority of previous studies have commonly shown to be composed of two or three lower order factors (Silverman et al., 1999, 2003). Our findings point to a few possibilities with regard to the construct of AS within our sample: that the 9-item ‘physical concerns’ measure can be used to measure an aspect of AS (i.e., ‘physical concerns’) as it has originally been operationalized; that ‘social concerns’ and ‘control concerns’ are not particularly salient in our sample and/or that the AS construct may need to be operationalized in a different way. To address the aforementioned, further research within the South African context is required to reveal whether results similar to ours are determined and whether the AS construct in comparable samples can be further clarified.

### Limitations and Future Studies

A few study limitations deserve mention. Firstly, measurement invariance of the CASI through cultural groups could not be established as the study design allowed for the AS construct to be assessed in only one multicultural South African sample. Secondly, responses to the self-report items may have been influenced by both the interpretation and complexity of items, influencing the validity of the self-report measures. Thirdly, owing to the use of self-report data, responses on the CASI may have been over- or under-reported. Fourthly, no structured or semi-structured interviews were conducted to assess anxiety disorders in our sample, which may have inflated scores. Lastly, in the analysis, the responses on the CASI’s 3-point ordinal scale were treated as continuous. In spite of these limitations, this study makes a useful contribution to the literature on the construct of AS in a large, representative sample of predominantly non-Caucasian youth in a multi-cultural setting and provides the initial step in investigating the validity of this construct in South African youth.

Recommendations for future research include (1) a follow-up study to this study that allows for the 18-item CASI measure to be re-administered in this multi-cultural sample of youth so as to assess the measurement invariance of the CASI (Vandenberg, 2002), and whether it requires adaptation for this population; and (2) subsequent replication studies in multicultural community and clinical samples, in other low- and/or middle-income country (LMIC) settings to provide further insight into the construct of AS in these samples. This will aid in determining the validity of the AS construct among youth in LMIC contexts according to ethnicity, socio-economic and clinical status.

## Conclusion

In sum, the one-factor model, derived from the 18-item CASI, consisting predominantly of physical concerns, seemed to provide the best fit to our data. A 9-item ‘physical concerns’ factor, derived after deletion of items, did not improve on the amount of variance extracted from the 18-item one-factor model. In terms of gender, a one-factor model was suited to both males and females and factor loadings were similar across gender. Item difficulty and complexity may have influenced participants’ responses and thus may have contributed to our findings of relatively low factor loadings and levels of variance extracted. As such, we recognize the limitations of the use of the CASI in our sample in its current form and suggest that the CASI be administered in other multi-cultural samples of youth in South Africa so as to provide further clarification of the AS construct in such samples.

## Author Contributions

LM: substantial contributions to the acquisition of data, interpretation of data, drafting and revision of manuscript, final approval of manuscript to be submitted.

MK: substantial contribution to the interpretation of data for the work, revising the work critically for important intellectual content, final approval of manuscript to be submitted.

SS: substantial contributions to the conception and design of the work, revising the work critically for important intellectual content, final approval of manuscript to be submitted.

LM, MK, and SS: agreement to be accountable for all aspects of the work in ensuring that questions related to the accuracy or integrity of any part of the work are appropriately investigated and resolved.

## Conflict of Interest Statement

The authors declare that the research was conducted in the absence of any commercial or financial relationships that could be construed as a potential conflict of interest.
